# A Reduced Dose Whole Virion Aluminum Adjuvanted Seasonal Influenza Vaccine Is Immunogenic, Safe, and Well Tolerated in Pediatric Patients †

**DOI:** 10.3390/v13030500

**Published:** 2021-03-18

**Authors:** Zoltan Vajo, Gergely Balaton, Peter Vajo, Peter Torzsa

**Affiliations:** 1 Department of Family Medicine, Semmelweis University Medical School, 1125 Budapest, Hungary; torzsa.peter@med.semmelweis-univ.hu; 2 Department of Pediatric Dentistry, Semmelweis University Medical School, 1088 Budapest, Hungary; gergelybalaton@gmail.com; 3 Clinical Center, University of Debrecen, 4032 Debrecen, Hungary; vape5th@icloud.com

**Keywords:** influenza, vaccine, children, immunogenicity, reactogenity

## Abstract

Background: Data suggest that pediatric patients might react differently to influenza vaccination, both in terms of immunity and side effects. We have recently shown that using a whole virion vaccine with aluminum phosphate adjuvants, reduced dose vaccines containing 6 µg of viral hemagglutinin (HA) per strain are immunogenic, and well tolerated in adult and elderly patients. Here we show the results of a multicenter clinical trial of pediatric patients, using reduced doses of a new, whole virion, aluminum phosphate adjuvanted vaccine (FluArt, Budapest, Hungary). Methods: A total of 120 healthy volunteers were included in two age groups (3–11 years, receiving 3 µg of HA per strain, and 12–18 years, receiving 6 µg of HA per strain). We used hemagglutination inhibition testing to assess immunogenicity, based on EMA and FDA licensing criteria, including post/pre-vaccination geometric mean titer ratios, seroconversion and seropositivity rates. Safety and tolerability were assessed using CHMP guidelines. Results: All subjects entered the study and were vaccinated (ITT population). All 120 subjects attended the control visit on Day 21 (PP population). All immunogenicity licensing criteria were met in both age groups for all three vaccine virus strains. No serious adverse events were detected and the vaccine was well tolerated by both age groups. Discussion: Using a whole virion vaccine and aluminum phosphate adjuvants, a reduction in the amount of the viral hemmaglutinin is possible while maintaining immunogenicity, safety and tolerability in pediatric and adolescent patients.

## 1. Introduction

### 1.1. Scientific Background and Explanation of Rationale

Influenza, though often unrecognized, is a major cause of illness and hospitalization in children [1]. In addition, children play an important role in the transmission of influenza. Therefore, vaccinating this population is important not only for direct but also indirect protection for the wider population. Some data suggest that pediatric patients might react differently to influenza vaccination, both in terms of immunity and side effects [2,3]. Conventional (nonadjuvanted) trivalent inactivated influenza vaccines are less effective in children than in adults, possibly because the immune responses are less robust than those of adults [4,5,6]. Evidence from several studies indicates that children aged 6 months through 8 years require 2 doses of influenza vaccine (administered a minimum of 4 weeks apart) during their first season of vaccination for optimal protection [7,8].

We have previously shown that a monovalent, prepandemic influenza vaccine (A H5N1) is immunogenic, safe and well tolerated in children and adolescents, when formulated with inactivated whole virion, and adjuvanted by aluminum phosphate, even with a smaller than usual amount of active ingredient, 6 µg of viral HA/strain [9]. Furthermore, when studying seasonal trivalent vaccines, we have recently found that using an inactivated whole virion vaccine with aluminum phosphate adjuvant, reduced dose vaccines containing 6 µg of viral HA per strain are immunogenic, safe and well tolerated in adult and elderly patients, and thus, allow dose sparing and increased production capacity [10].

### 1.2. Specific Objectives or Hypotheses

In the present trial, we studied a reduced dose, trivalent seasonal influenza vaccine, produced by the above principle (i.e., inactivated, whole virion, aluminum phosphate adjuvanted), in pediatric populations, in terms of immunogenicity, safety and tolerability, based on criteria by the U.S. Food and Drug Administration (Silver Spring, MD, USA), as well as the European Medicines Agency (Amsterdam, The Netherlands) [11,12].

## 2. Methods

### 2.1. Description of Trial Design, Settings and Locations

This was a multicenter, stratified (for age: 3–11 years and 12–18 years old), prospective, uncontrolled study, conducted in the European Union (Budapest, Hungary) at 2 centers, between 18 October 2014 (enrollment of the first participant) and 9 December 2014 (last visit of the last subject participating). No changes were made to methods after trial commencement. The study protocol, the patient information sheet, the informed consent form and other appropriate study-related documents were reviewed by the Ethics Committee for Clinical Pharmacology of the Medical Research Council (ETT KFEB), and approved by the National Institute of Pharmacy of Hungary (OGYI) in accordance with domestic legal requirements. The study was conducted in accordance with the rules of good clinical practice. The conditions were in compliance with the Declaration of Helsinki [13]. The study was registered under the European Union Drug Regulating Authorities Clinical Trials Database, (EudraCT), Number 2013-003449-40.

### 2.2. Eligibility Criteria for Participants

Participants were recruited by their primary care physicians. Subjects eligible for enrolment into this study were: male and female patients aged 3–18years, with power of attorneys mentally competent, able to understand and comply with all study requirements, able to give written informed consent prior to initiation of study procedures, in good health (as determined by the investigator on the basis of medical history and examination) and stable medical condition.

Exclusion criteria included: pregnancy, hypersensitivity to eggs, chicken protein, thiomersal, formaldehyde, gentamycin, ciprofloxacin, neomycin or any other components of the vaccine, history of anaphylactic shock or neurological symptoms following administration of any vaccine; history of Guillain-Barre syndrome; malignant tumor, autoimmune disease, advanced arteriosclerotic disease, complicated diabetes mellitus, acute or progressive hepatic disease, acute or progressive renal disease, congestive heart failure; immunosuppressive therapy within 36 months prior to vaccination; concomitant corticosteroid therapy (including high-dose inhaled corticosteroids); receiving immunostimulants; receipt of immunoglobulin, blood products and/or plasma derivates within 3 months prior to vaccination; HIV, HBV or HCV infection; fever of >37 °C within 3 days prior to vaccination; vaccine therapy within 4 weeks; influenza vaccination within 6 months prior to vaccination; experimental drug therapy within 4 weeks prior to vaccination; concomitant participation in another clinical study.

### 2.3. Vaccines

The vaccine was a whole virus, aluminum phosphate adjuvanted, trivalent vaccine against seasonal influenza containing 6 µg of HA per virus strain. The influenza viruses included in the vaccine were grown in embryonated hen eggs, inactivated by formaldehyde, purified and concentrated, and absorbed on aluminum phosphate gel, as described in detail elsewhere [14]. The virus strains were chosen according to the WHO and European Union recommendations for the seasonal influenza vaccine composition for the season of 2014/15 as follows: reassortant virus NYMC X-179A, which is derived from A/California/7/2009(H1N1)pdm09, reassortant virus NYMC X-223A, which is derived from A/Texas/50/2012(H3N2), and B/Massachusetts/2/2012-like virus (Yamagata lineage) [15]. The hemagglutinin (HA) content was determined before the addition of the aluminum phosphate adjuvant by a single radial immunodiffusion test, using reagents supplied by NIBSC (Herts, UK), as reported previously [16,17] Purity was evaluated by endotoxin content, which was <0.05 IU/dose, and the amount of ovalbumin, which was <5 ng/dose. Both values are much lower than the concentrations considered acceptable by the European Pharmacopoeia, which are 100 IU and 1000 ng/human dose, respectively [18]. Aluminum phosphate was added as adjuvant, in the amount of 0.31 mg Al/ampoule, meeting the requirements of the European Pharmacopoeia [18]. The vaccine was manufactured according to Good Manufacturing Practice GMP requirements by Fluart LTD (Budapest, Hungary).

### 2.4. Interventions

Subjects were assigned to one of the following age groups: participants aged 3–11 years received one 0.25 mL intramuscular injection of the investigational vaccine, containing 3 µg per virus strain, while patients 12–18 years of age were given one 0.5 mL injection of the vaccine, containing 6 µg per strain. Doses of the study vaccine administered were determined based on previous clinical trials by our group [9].

All subjects were observed for 30 min after vaccination for any immediate reactions and were instructed to complete a diary card to record local (ecchymosis, erythema, induration, swelling and pain at injection site) and systemic reactions (chills, malaise, myalgia, arthralgia, headache, sweating, fatigue and potential indicators of oculo-respiratory syndrome), and axillary temperature starting on the day of vaccination and for each of the 7 days following the immunization. All adverse events were collected from the day of vaccination until day 120. All adverse events necessitating a physician’s visit or consultation and/or leading to premature study discontinuation and all serious adverse events were collected throughout the entire trial and data were reconciled at study termination visit.

Baseline evaluations on Day 0 included obtaining demographic data, medical history, and performing a complete physical examination. Blood samples were drawn for baseline hemagglutination inhibition test (HI) for all three vaccine virus strains. Serum antibody titers against the vaccine virus strain were measured by HI, using chicken red blood cells (CRBC) and following standard procedures, as recommended by the WHO [19]. Briefly, HI was assayed in 96-well microtiter plates. Serum was treated with receptor-destroying enzyme (Denka Seiken Co., Tokyo, Japan) overnight at 37 °C, heat-inactivated at 56 °C for 45 min, diluted 1:10 with sterile PBS, and tested by HI assay with 0.5% packed CRBC. Antibody titrations were done in duplicate; pre- and post-vaccination sera were titrated simultaneously. The titer assigned to each sample was the geometric mean of two independent determinations. For the purpose of calculation, any HI result <10 was expressed as 5. Sera were titrated in two independent determinations. Each determination included two parallel measurements. Geometric mean of the two parallel, then geometric mean of the two independent determinations were calculated. On day 21, a medical history and the list of medications used during the days since the last visit were obtained, physical examination was performed, and blood samples were drawn for HI.

### 2.5. Pre-Specified Primary and Secondary Outcome Measures

The primary outcome measure was the immungenicity of the reduced dose vaccine containing 3 or 6 µg of HA per strain as measured by HI test 21 days after vaccination. We used HI testing to assess immunogenicity, based on EMA and FDA licensing criteria, including post/pre-vaccination geometric mean titer ratios, seroconversion and seropositivity rates [11,12].

The safety objective was to evaluate the tolerability and safety of the administration of the vaccine doses containing either 3 or 6 µg of HA of seasonal A/H1N1, A/H3N2 and B influenza antigens as determined by the licensing requirements of the European Union [11]. No changes were made to the protocol after the trial commenced. The study medication was provided by the manufacturer, Fluart LTD, Budapest, Hungary. The source had no role in the conduct of the study or the preparation of the manuscript.

### 2.6. Sample Size Determination

A sample size of at least 50 evaluable participants per group was selected based on CHMP licensing criteria [11] and based on a log10 standard deviation of Geometric Mean Titer Ratios GMTRs of 0.47, estimated conservatively upon data previously reported in clinical trials of this vaccine [10,20].

It was estimated that a number of 50 subjects would be necessary in each age group to have 80% power for detection of a minimum expected GMTR of 3.125-fold by Day 21–28 post-vaccination in case of all of the investigated influenza virus strains. This sample size was thus sufficient for detecting with 80% power a minimum expected seroconversion rate of 44.54% and a minimum expected seroprotection rate of 76.68%, for all of the investigated influenza virus strains. Considering an approximately 17% of drop-out (or one participant out of six), a total of 120 healthy volunteers were included, (sixty (60) subjects in each age group) in order to achieve at least a total of 100 evaluable subjects, meaning 50 subjects in each age group).

### 2.7. Statistical Methods Used

Descriptive statistics (mean, standard deviation, median, minimum and maximum) for age at enrolment were calculated overall and by age group. Distributions of subjects by gender and previous influenza vaccination status were summarized overall and by age group.

HI antibody titers were determined at baseline, and at Day 21. HI titers were used to calculate seroconversion rates, seroprotection rates, and increase in GMTRs, according to EMEA and FDA guidelines [11,12]. Distributions of the logarithms of Day 0 and Day 21 titers in each subgroup were visualized with normal quantile-quantile plot to ensure normality. The Primary immunogenicity objective was to evaluate the immunogenicity of the reduced dose influenza vaccine containing 3 or 6 micrograms of HA according to the applicable European Union (CHMP) and United States (FDA) requirements [11,12]. During the assessment, the following serological parameters were assessed: (i) seroconversion or ≥4-fold increase in HI antibody titer in the participants; (ii) post/pre vaccination GMTRs; and (iii) percentage of subjects achieving an HI titer of >40 or greater after immunization.

In order to confirm protective immunogenicity, at least one of the following three requirements had to be met in both aged groups: the rate of seroconversions or significant increase in antihaemagglutinin antibody titre is >40%; mean geometric increase >2.5; rate of subjects achieving an HI titre ≥40 >70% of subjects.

All calculations are made with the R programming language for statistics version 2.15.0. No interim analyses were performed. No changes in the outcomes were made after the trial commenced. No interim analyses were performed.

## 3. Results

A total of 120 healthy subjects (males and females) were selected for inclusion in the study, and screened prior to vaccination. All subjects screened were found to be eligible, entered the study and were vaccinated (ITT population). All of the 120 subjects (60 subjects per age group) attended the control visit at Days 21–28, and their data were available and evaluated at Days 21–28 (PP population).

The baseline characteristics, including age and gender distribution are summarized for the two vaccination groups in Table 1. A total of 5 subjects received seasonal influenza vaccination in the previous season. Individual HA titers are available upon request.

Both vaccine doses fulfilled all applicable immunogenicity licensing criteria by EMA and FDA guidelines for the age groups investigated for all three vaccine virus strains (Table 2).

Administration of the vaccine was well tolerated by the study subjects in both age groups. All possibly or probably related AEs occurred during the study were mild (37 cases) or moderate (one case, headache), and recovered without sequels. No serious and no severe possibly or probably related adverse event was observed. The vaccine proved to be safe at both doses, no vaccine-related clinically significant changes in the physical condition or vital signs of the volunteers were observed (Table 3).

## 4. Discussion

### 4.1. Interpretation

Most currently available influenza vaccines are split virion or subunit vaccines, without adjuvants, containing 15 µg of HA per virus strain. However, a renewed interest in whole virion vaccines has recently developed, at least in cases when increased immunogenicity or cross protection was desired, such as with emerging new strains or during pandemics [17,21,22,23]. Similarly, in scenarios when increased immunogenicity is needed, as in elderly patients, adjuvanted influenza vaccines have been developed and successfully used [10,24]. Aluminum salts, such as aluminum hydroxide, aluminum phosphate, and aluminum potassium sulfate have been used safely in vaccines for more than 70 years, including currently licensed vaccines, such as Tdap (Tetanus, Diphtheria, Pertussis), MenB (Serogroup B Meningococcus), Japanese encephalitis, HPV (Human papillomavirus) and others [25,26].

We have previously shown that using inactivated whole virion vaccines and aluminum phosphate adjuvants, a reduction in the amount of the viral hemmaglutinin antigen is possible, while maintaining adequate immunogenicity, safety and tolerability profiles in adult and elderly patients in case of seasonal trivalent influenza vaccines (10, 20). We have found similar efficacy with monovalent pandemic H1N1 and prepandemic H5N1 whole virion, aluminum adjuvanted influenza vaccines in adult, elderly, and pediatric patients [9,17,27]. As of protection, we previously found in animal studies that the same vaccine dose and formulation is protective in Chinese painted quails (Coturnix chinensis) against Influenza A infection [28].

Vaccine doses for children 3–18 years of age for seasonal influenza are currently recommended at 15–30 µg/strain (i.e., one or two doses of the 15 µg, vaccines) based on age and previous vaccination status [29]. In the present trial, we found that using a trivalent seasonal, inactivated whole virion influenza vaccine adjuvanted by aluminum phosphate was immunogenic, safe and well tolerated in for all three virus strains in pediatric (ages 3–11 years) and adolescent (ages 12–18 years) patients, with doses as low as 3 and 6 µg respectively. Since vaccine shortages occur frequently, using reduced dose vaccines, which allow increased production capacity, are highly desirable [30].

We believe that the acceptable reactogenity profile we have seen in the present and previous trials with whole virion influenza vaccines can be at least in part explained by the endotoxin content of the vaccine being well below the values permitted by the European Pharmacopia.

### 4.2. Limitations and Generalizability

We did not study children younger than 3 years of age, and thus, we do not know if our results can be generalized for them. Since we did not test the whole virion vaccine without adjuvants, we are unable to determine whether the immunogenicity results seen in the present trial at reduced doses were due to using a whole virion vaccine, using an adjuvant, or both. Our study recruited only white Caucasian subjects. Although there are currently no licensing requirements to indicate such, there is at least some published evidence that there might be race-related differences in the antibody response to influenza vaccination [31]. Our goal was to determine whether a reduced dose vaccine is able to fulfill licensing requirements. Hence, we did not investigate non-inferiority to a licensed vaccine.

## Figures and Tables

**Table 1:** Demographic information of the participants.

Participants	No.	Mean Age ± SD (Years)	Median (Years)	Min. (Years)	Max. (Years)
Total	120	11.16 ± 3.89	11.50	3.00	17.00
Pediatric (3–11 years)	60	7.93 ± 2.65	8.00	3.00	11.00
Adolescent (12–18 years)	60	14.38 ± 1.54	14.00	12.00	17.00
Males	67	10.88 ± 3.92	11.00	3.00	17.00
Females	53	11.51 ± 3.86	12.00	3.00	17.00

**Table 2:** Immunogenicity licensing criteria and study results.

Age Group	Strain	Critetion for Licensing	Requirement	Results	Outcome
3–11 years	H1N1	Seroconversion	≥40%	61.67%	fulfilled
		GMT ratio	≥2.5	4.337	fulfilled
		Seroprotection	≥70%	91.67%	fulfilled
	H3N2	Seroconversion	≥40%	58.33%	fulfilled
		GMT ratio	≥2.5	4.982	fulfilled
		Seroprotection	≥70%	95.00%	fulfilled
	B	Seroconversion	≥40%	66.67%	fulfilled
		GMT ratio	≥2.5	4.262	fulfilled
		Seroprotection	≥70%	80.00%	fulfilled
12–18 years	H1N1	Seroconversion	≥40%	66.67%	fulfilled
		GMT ratio	≥2.5	4.702	fulfilled
		Seroprotection	≥70%	96.67%	fulfilled
	H3N2	Seroconversion	≥40%	61.67%	fulfilled
		GMT ratio	≥2.5	5.993	fulfilled
		Seroprotection	≥70%	95.00%	fulfilled
	B	Seroconversion	≥40%	70.00%	fulfilled
		GMT ratio	≥2.5	4.757	fulfilled
		Seroprotection	≥70%	86.67%	fulfilled

**Table 3:** Distribution of possibly or probably related adverse events by age group.

System of Concern	Symptom	Age Group		Total
		3–11	12–18	
General disorders and administration site conditions	Vaccination site pain	21	17	38
	Vaccination site erythema	7	2	9
	Vaccination site induration	3	5	8
	Vaccination site swelling	7	2	9
	Vaccination site hematoma	2	-	2
	Rigors	-	1	1
	Malaise	1	7	8
	Pyrexia	1	2	3
Musculoskeletal and connective tissue disorders	Myalgia	4	8	12
Gastrointestinal disorders	Vomiting	-	1	1
	Abdominal pain	-	1	1
Nervous system disorders	Headache	-	4	4

## Data Availability

The data presented in this study are available on request from the corresponding author. The data are not publicly available due to privacy.

## References

[1] Poehling K.A., Edwards K.M., Weinberg G.A., Szilagyi P., Staat M.A., Iwane M.K., Bridges C.B., Grijalva C.G., Zhu Y., Bernstein D.I. (2006). The Underrecognized Burden of Influenza in Young Children. N. Engl. J. Med..

[2] Wilkins A.L., Kazmin D., Napolitani G., Clutterbuck E.A., Pulendran B., Siegrist C.-A., Pollard A.J. (2017). AS03- and MF59-Adjuvanted Influenza Vaccines in Children. Front. Immunol..

[3] Trogstad L., Bakken I.J., Gunnes N., Ghaderi S., Stoltenberg C., Magnus P., Håberg S.E. (2017). Narcolepsy and hypersomnia in Norwegian children and young adults following the influenza A(H1N1) 2009 pandemic. Vaccine.

[4] Grohskopf L.A., Sokolow L.Z., Broder K.R., Walter E.B., Bresee J.S., Fry A.M., Jernigan D.B. (2017). Prevention and Control of Seasonal Influenza with Vaccines: Recommendations of the Advisory Committee on Immunization Practices—United States, 2017–2018 Influenza Season. MMWR Recomm. Rep..

[5] Jefferson T., Rivetti A., Harnden A., Di Pietrantonj C., Demicheli V. (2012). Vaccines for preventing influenza in healthy children. Cochrane Database Syst. Rev..

[6] Pichichero M.E. (2014). Challenges in vaccination of neonates, infants and young children. Vaccine.

[7] Neuzil K.M., Jackson L.A., Nelson J., Klimov A., Cox N., Bridges C.B., Dunn J., DeStefano F., Shay D. (2006). Immunogenicity and reactogenicity of 1 versus 2 doses of trivalent inactivated influenza vaccine in vaccine-naive 5-8-year-old children. J. Infect. Dis..

[8] Ritzwoller D.P., Bridges C.B., Shetterly S., Yamasaki K., Kolczak M., France E.K. (2005). Effectiveness of the 2003–2004 Influenza Vaccine Among Children 6 Months to 8 Years of Age, With 1 vs 2 Doses. Pediatrics.

[9] Vajo Z., Kosa L., Szilvasy I., Pauliny Z., Bartha K., Visontay I., Kis A., Tarjan I., Rozsa N., Jankovics I. (2008). Safety and Immunogenicity of a Prepandemic Influenza A (H5N1) Vaccine in Children. Pediatr. Infect. Dis. J..

[10] Vajo Z., Kalabay L., Vajo P., Balaton G., Rozsa N., Torzsa P. (2019). Licensing the first reduced, 6 µg dose whole virion, aluminum adjuvanted seasonal influenza vaccine—A randomized-controlled multicenter trial. Vaccine.

[11] European Committee for Proprietary Medicinal Products Note for Guidance on Harmonization of Requirements for Influenza Vaccines. 12 March 1997 (CPMP/BWP/214/96). www.ema.europa.eu/docs/en_GB/document_library/Scientific_guideline/2009/09/WC500003945.pdf.

[12] Food and Drug Administration, Center for Biologics Evaluation and Research (2007). Guidance for Industry. Clinical Data Needed to Support the Licensure of Seasonal Inactivated Influenza Vaccines. U.S. Department of Health and Human Services. www.fda.gov/downloads/BiologicsBloodVaccines/GuidanceComplianceRegulatoryInformation/Guidances/Vaccines/ucm091990.pdf.

[13] World Medical Association Declaration of Helsinki. www.wma.net/what-we-do/medical-ethics/declaration-of-helsinki/doh-oct2000/.

[14] Vajo Z., Kosa L., Szilvasy I., Pauliny Z., Bartha K., Visontay I., Jankovics M., Kis A., Jankovics I. (2008). Yearly licensing studies from 1997 to 2007 of the inactivated whole virus seasonal influenza vaccine fluval—A useful approach to pandemic vaccine development even in less well developed countries?. Influenza Other Respir. Viruses.

[15] Committee for Medicinal Products for Human Use, BWP Ad-hoc Influenza Working Group (2014). EU Recommendations for the Seasonal Influenza Vaccine Composition for the Season 2014/2015. www.ema.europa.eu/en/documents/other/biologics-working-party-ad-hoc-influenza-working-group-european-union-recommendations-seasonal/2015_en.pdf.

[16] Wood J.M., Schild G.C., Newman R.W., Seagroatt V. (1977). An improved single-radial-immunodiffusion technique for the assay of influenza haemagglutinin antigen: Application for potency determinations of inactivated whole virus and subunit vaccines. J. Biol. Stand..

[17] Vajo Z., Wood J., Kosa L., Szilvasy I., Paragh G., Pauliny Z., Bartha K., Visontay I., Kis A., Jankovics I. (2009). A Single-Dose Influenza A (H5N1) Vaccine Safe and Immunogenic in Adult and Elderly Patients: An Approach to Pandemic Vaccine Development. J. Virol..

[18] Bouin A.-S., Wierer M. (2014). Quality standards of the European Pharmacopoeia. J. Ethnopharmacol..

[19] WHO Global Influenza Surveillance Network (2011). Manual for the Laboratory Diagnosis and Virological Surveillance of Influenza.

[20] Vajo Z., Balaton G., Vajo P., Kalabay L., Erdman A., Torzsa P. (2017). Dose sparing and the lack of a dose-response relationship with an influenza vaccine in adult and elderly patients—A randomized, double-blind clinical trial. Br. J. Clin. Pharmacol..

[21] Budimir N., Huckriede A., Meijerhof T., Boon L., Gostick E., Price D.A., Wilschut J., De Haan A. (2012). Induction of Heterosubtypic Cross-Protection against Influenza by a Whole Inactivated Virus Vaccine: The Role of Viral Membrane Fusion Activity. PLoS ONE.

[22] Furuya Y. (2011). Return of inactivated whole-virus vaccine for superior efficacy. Immunol. Cell Biol..

[23] Fazekas G., Martosne-Mendi R., Jankovics I., Szilvasy I., Vajo Z. (2008). Cross-Reactive Immunity to Clade 2 Strains of Influenza Virus A Subtype H5N1 Induced in Adults and Elderly Patients by Fluval, a Prototype Pandemic Influenza Virus Vaccine Derived by Reverse Genetics, Formulated with a Phosphate Adjuvant, and Directed to Clade 1 Strains. Clin. Vaccine Immunol..

[24] Nicolay U., Heijnen E., Nacci P., Patriarca P.A., Leav B. (2019). Immunogenicity of aIIV3, MF59-adjuvanted seasonal trivalent influenza vaccine, in older adults ≥65 years of age: Meta-analysis of cumulative clinical experience. Int. J. Infect. Dis..

[25] Centers for Disease Control Vaccine Excipient Summary. Excipients Included in U.S. Vaccines, by Vaccine. www.cdc.gov/vaccines/pubs/pinkbook/downloads/appendices/B/excipient-table-2.pdf.

[26] Keith L., Jones D., Chou C.-H. (2002). Aluminum toxicokinetics regarding infant diet and vaccinations. Vaccine.

[27] Vajo Z., Tamas F., Sinka L., Jankovics I. (2010). Safety and immunogenicity of a 2009 pandemic influenza A H1N1 vaccine when administered alone or simultaneously with the seasonal influenza vaccine for the 2009–2010 influenza season: A multicentre, randomised controlled trial. Lancet.

[28] Sarkadi J., Jankovics M., Kis Z., Skare J., Fodor K., Gonczol E., Visontai I., Vajo Z., Jankovics I. (2013). Protection of Chinese painted quails (Coturnix chinensis) against a highly pathogenic H5N1 avian influenza virus strain after vaccination. Arch. Virol..

[29] Committee on Infectious Diseases (2019). Recommendations for Prevention and Control of Influenza in Children, 2019–2020. Pediatrics.

[30] Kempe A., Daley M.F., Stokley S., Crane L.A., Beaty B.L., Barrow J., Babbel C., Dickinson L.M., Steiner J.F., Berman S. (2007). Impact of a Severe Influenza Vaccine Shortage on Primary Care Practice. Am. J. Prev. Med..

[31] Kurupati R., Kossenkov A., Haut L., Kannan S., Xiang Z., Li Y., Doyle S., Liu Q., Schmader K., Showe L. (2016). Race-related differences in antibody responses to the inactivated influenza vaccine are linked to distinct pre-vaccination gene expression profiles in blood. Oncotarget.

